# Perinatal and Parental Predictors of Wheezing in the First Year of Life: A Saudi Arabian Birth Cohort Study

**DOI:** 10.3390/healthcare14131996

**Published:** 2026-07-05

**Authors:** Nasser S. Alharbi, Lana A. Shaiba, Mona Philby, Ebtesam Almutairi, Fahad Alsohime, Mohamad-Hani Temsah

**Affiliations:** 1 Department of Pediatrics, College of Medicine, King Saud University, Riyadh 12372, Saudi Arabia; lshaiba@ksu.edu.sa (L.A.S.); falsohime@ksu.edu.sa (F.A.); temsah1@yahoo.com (M.-H.T.); 2 Department of Pediatrics, King Saud University Medical City, King Saud University, Riyadh 12372, Saudi Arabia; monaphilby@yahoo.com (M.P.); ebtesam.sayer@gmail.com (E.A.)

**Keywords:** infant wheezing, recurrent wheezing, NICU, parental atopy, Saudi Arabia, birth cohort

## Abstract

**Background:** Infant wheezing is a common respiratory condition with a significant healthcare burden, yet data from the Middle East remain limited. This study aimed to identify predictors of any wheezing and recurrent wheezing during the first year of life in a Saudi Arabian birth cohort. **Methods:** This retrospective birth cohort study included infants born at King Saud University Medical City, Riyadh, in 2020. Data were collected from electronic medical records and a structured parental questionnaire administered via WhatsApp at 12 months of age, assessing wheezing episodes, parental atopy, household smoking, pets, home humidity, and nearby pollution sources. Any wheezing was defined as ≥1 wheezing episode during the first year, and recurrent wheezing as ≥3 episodes. Variables with *p* < 0.25 in univariable logistic regression were entered into multivariable models; adjusted odds ratios (aOR) with 95% confidence intervals are reported. **Results:** Of 594 infants, 135 (22.7%) experienced any wheezing and 85 (14.3%) had recurrent wheezing. NICU admission was independently associated with both any wheezing (aOR 2.65, 95% CI 1.49–4.73; *p* < 0.001) and recurrent wheezing (aOR 2.34, 95% CI 1.21–4.56; *p* = 0.012). Parental allergic rhinitis was independently associated with both outcomes (any wheezing: aOR 1.55, 95% CI 1.01–2.37; recurrent wheezing: aOR 1.67, 95% CI 1.00–2.78), while parental eczema was specifically associated with recurrent wheezing (aOR 1.74, 95% CI 1.00–3.03). **Conclusions:** NICU admission and parental atopy were associated with infant wheezing in this cohort. These findings provide region-specific data from Saudi Arabia but should be regarded as hypothesis-generating and require confirmation in prospective multicentre studies before informing clinical follow-up strategies.

## 1. Introduction

Infant wheezing is one of the most common respiratory conditions in early childhood. Approximately one-third of children experience at least one wheezing episode during the first three years of life [1]. The cumulative incidence of wheezing by 12 months of age varies across regions, ranging from 33.7% in Italy and 34.4% globally to 40.1% in the United Kingdom and 47% in Latin America [2,3,4]. Recurrent wheezing, typically defined as three or more episodes within a year, affects 10–17% of infants worldwide [2,5]. Infant wheezing, particularly recurrent episodes, imposes a substantial burden on healthcare systems and families. Among infants with recurrent wheezing, 72.7% require at least one emergency department visit and 29.7% are hospitalized during the first year of life [5]. Recurrent wheezing is also associated with an increased risk of pneumonia hospitalization, affecting 20.1% of infants with recurrent wheezing compared to 7.9% of the general infant population [5]. Furthermore, medication utilization is high among affected infants, with nearly half receiving inhaled corticosteroids and over three-quarters prescribed antibiotics [5]. These findings underscore the clinical and economic significance of identifying infants at risk for wheezing early in life.

Viral infections are the predominant cause of infant wheezing. Respiratory syncytial virus (RSV) is the most common pathogen causing bronchiolitis in infants younger than 12 months, representing a major cause of morbidity globally [1,6]. In contrast, rhinovirus becomes the dominant viral etiology in children aged 12–24 months [1,7]. Rhinovirus is involved in more than 50% of upper respiratory tract infections and accounts for 20–40% of bronchiolitis cases in infants under one year, rising to 50% among hospitalized children under three years [7]. Early viral wheezing is strongly associated with subsequent asthma development. In the INSPIRE birth cohort, 20.75% of infants with RSV infection in the first year of life developed asthma by age 5, while a Finnish cohort reported that 53% of children hospitalized for viral wheezing before age 2 had asthma by young adulthood [1,6]. Notably, rhinovirus-associated wheezing may confer a higher risk of future asthma than RSV, possibly due to a more pronounced Th2-mediated immune response [7]. These findings underscore the importance of identifying infants with early viral wheezing who may be at risk for persistent respiratory morbidity.

Multiple risk factors for infant wheezing have been identified in birth cohort studies worldwide. Parental asthma is among the strongest predictors, with maternal asthma associated with an aOR of 4.03 (95% CI 2.21–7.37) for persistent wheezing and paternal asthma with an aOR of 2.11 (95% CI 1.35–3.30) [8,9,10]. Parental allergic rhinitis and eczema also increase wheezing risk, with reported ORs ranging from 1.13 to 1.83 [9,11,12]. Among child factors, male sex is consistently associated with higher wheezing risk (OR 1.41–1.50), while preterm birth (RR 1.2, 95% CI 1.0–1.3) and low birth weight (RR 1.28, 95% CI 1.04–1.58) also confer increased susceptibility [10,11,13,14]. Child atopic dermatitis is associated with earlier wheezing onset (aHR 1.25, 95% CI 1.12–1.39) [9,12]. Environmental exposures play a significant role; tobacco smoke exposure increases wheezing risk (IRR 1.70–2.09), while daycare attendance is associated with both transient and recurrent wheezing (aOR 1.63–2.16) [8,9,11,13,15]. Conversely, breastfeeding for more than six months appears protective (aOR 0.52, 95% CI 0.39–0.75) [11,12]. Having siblings and living in damp housing have also been linked to increased wheezing risk [9,15].

Despite the growing body of literature on infant wheezing risk factors, data from the Middle East, particularly Saudi Arabia, remain limited. A recent meta-analysis reported an asthma prevalence of 16.57% among Saudi children, yet birth cohort studies examining early-life wheezing predictors in this region are scarce [16]. Furthermore, most existing cohorts have excluded infants admitted to the neonatal intensive care unit (NICU) to avoid confounding, resulting in a significant knowledge gap regarding wheezing risk in this high-risk population. Given the unique environmental exposures in the Gulf region, including high temperatures, indoor air conditioning use, sandstorms, and incense burning, region-specific data are needed to inform clinical practice and preventive strategies. Therefore, this study aimed to identify predictors of any wheezing and recurrent wheezing during the first year of life in a Saudi Arabian birth cohort.

## 2. Materials and Methods

### 2.1. Study Design and Setting

This retrospective birth cohort study was conducted at King Saud University Medical City (KSUMC), Riyadh, Saudi Arabia. All infants born between 1 January and 31 December 2020 were identified from hospital birth records. Families with valid contact information were invited to participate in a 12-month follow-up survey administered via WhatsApp. Birth and neonatal variables were obtained from electronic medical records documented at the time of birth, whereas wheezing outcomes and questionnaire-based exposures were ascertained retrospectively from parental report when the infant reached 12 months of age. Medical-record data were extracted only for families who completed the questionnaire; consequently, no birth-record information was available for non-responders. The participant flow, including the initial cohort, exclusions, response rate, and final analytic sample, is described in the Section 3.

### 2.2. Data Collection

Data were collected retrospectively from two sources:

1. Electronic Medical Records: Clinical data were extracted from hospital electronic medical records, including sex, birth weight, gestational age, mode of delivery, neonatal intensive care unit (NICU) admission, and physician-diagnosed infant atopic dermatitis.
2. A structured, parent-reported questionnaire: when a child reached one year of age, a structured, parent-reported questionnaire (Supplementary File S1) was administered to parents in a bilingual (Arabic–English) format via a survey link shared through WhatsApp, using their contact number recorded in the child’s medical record. Its core items were adapted, with written permission, from the previously validated EISL (International Study of Wheezing in Infants) questionnaire and were used as a structured, parent-reported instrument. Parents were asked to recall events that occurred during their child’s first 12 months of life. The initial message included a brief explanation of the study and its objectives. We ensured providing interactive messaging with parents to confirm understanding and to address any inquiries. A reminder message was sent to non-responders after a few days. The questionnaire included wheezing episodes during the first year of life, family history of allergic diseases (parental asthma, allergic rhinitis, and eczema), breastfeeding duration, daycare attendance, household smoking, home pets, nearby pollution sources, and maternal employment status.

### 2.3. Outcomes

Two binary outcomes were evaluated: (1) Any wheezing, defined as at least one parent-reported wheezing episode during the first year of life, and (2) recurrent wheezing, defined as three or more parent-reported wheezing episodes during the first year of life.

### 2.4. Ethical Considerations

The study was conducted in accordance with the Declaration of Helsinki and approved by the Institutional Review Board at King Saud University Medical City (IRB No. E-21-6480). Informed consent was obtained electronically as the first item of the survey, where parents were informed of the study’s objectives, the voluntary nature of participation, the right to withdraw, and the confidentiality of their data. All patient data were anonymized and handled in compliance with institutional and national data protection policies.

### 2.5. Statistical Analysis

Categorical variables were summarized as frequencies and percentages, while continuous variables were summarized using medians and interquartile ranges or means and standard deviations, as appropriate. Two binary outcomes were evaluated: Any wheezing during the first year of life and recurrent wheezing, defined as three or more wheezing episodes. Each outcome was analyzed separately using the same predefined analytical approach.

Univariate logistic regression analyses were performed to assess associations between candidate predictors and each wheezing outcome, with results reported as crude odds ratios (ORs), 95% confidence intervals (CIs), and *p* values. Only variables that demonstrated an association at a predefined screening threshold (*p* < 0.25) in univariate analysis were selected for inclusion in the multivariable models.

For each outcome, a clinically informed multivariable logistic regression model was constructed. All variables meeting the univariate screening criterion (*p* < 0.25) were entered simultaneously into the multivariable models and retained without further elimination, to minimize residual confounding and reduce the risk of overfitting. Multivariable models were fitted using complete-case analysis; the extent and source of missing data were examined to determine whether multiple imputation was required. The adequacy of events per variable was assessed, and multicollinearity among predictors was evaluated using variance inflation factors. Adjusted odds ratios (aORs) with corresponding 95% confidence intervals and *p* values are reported. Model calibration was assessed using the Hosmer–Lemeshow goodness-of-fit test. Statistical analyses were performed using SPSS software, version 31 (IBM Corp., Armonk, NY, USA), forest plots were generated using R software, version 2026.01.0 (RStudio, PBC, Boston, MA, USA). A two-sided *p* value < 0.05 was considered statistically significant.

## 3. Results

### 3.1. Study Population

**Figure 1:**
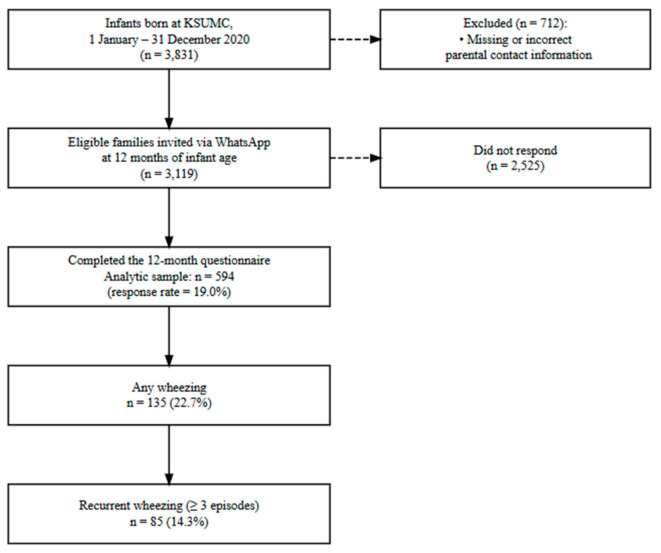
Participant flow diagram. Of 3831 infants born at King Saud University Medical City during 2020, 712 were excluded owing to missing or incorrect parental contact information. The remaining 3119 eligible families were invited to participate in the 12-month follow-up questionnaire. A total of 594 families responded (response rate 19.0%), constituting the final analytic sample.

Of 3831 infants identified from hospital birth records, 712 were excluded because of missing or incorrect parental contact information, leaving 3119 eligible families who were invited to participate. A total of 594 families completed the 12-month questionnaire (response rate 19.0%), constituting the analytic sample (Figure 1).

Of these, 135 children (22.7%) experienced any wheezing during the first year of life, and 85 (14.3%) had recurrent wheezing. The multivariable models were based on complete cases (n = 552); 42 infants (7.1%) were excluded owing to missing gestational age, while all other model variables were complete. Within this complete-case sample, there were 121 any-wheezing and 75 recurrent-wheezing events, corresponding to approximately 17 and 12 events per variable for the seven- and six-predictor models, respectively. Variance inflation factors were uniformly low in both models (all values < 1.5), indicating no problematic multicollinearity.

### 3.2. Predictors of Any Wheezing During Infancy

Baseline characteristics stratified by wheezing status are presented in Table 1.

In univariable logistic regression analyses (Table 2), NICU admission (OR 2.64, 95% CI 1.68–4.15; *p* < 0.001), daycare attendance (OR 2.30, 95% CI 1.14–4.63; *p* = 0.020), parental allergic rhinitis (OR 1.62, 95% CI 1.10–2.38; *p* = 0.014), child atopic dermatitis (OR 1.62, 95% CI 1.09–2.40; *p* = 0.016), and parental eczema (OR 1.61, 95% CI 1.03–2.49; *p* = 0.035) were associated with increased odds of wheezing during the first year of life and were therefore considered for inclusion in the multivariable model. Gestational age (OR 0.94, 95% CI 0.88–1.01; *p* = 0.069), and parental asthma (OR 1.45, 95% CI 0.89–2.37; *p* = 0.132) also met the pre-specified screening threshold (*p* < 0.25).

**Figure 2:**
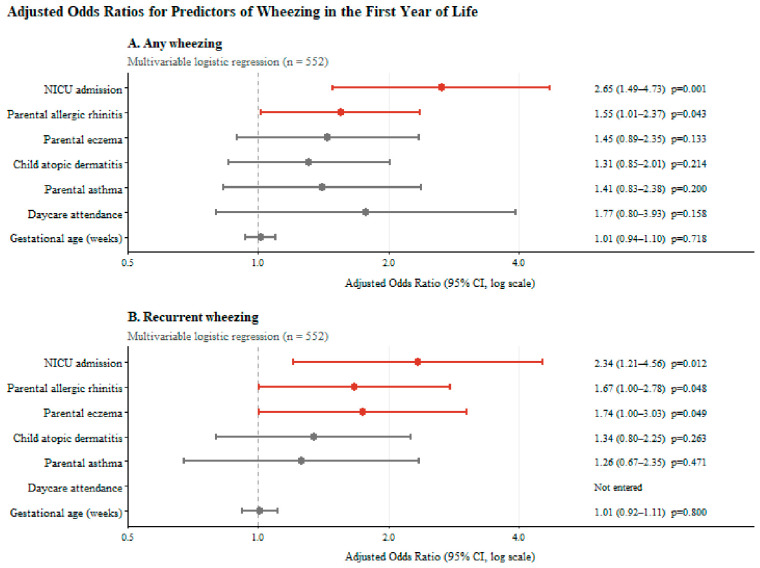
Forest plot of adjusted odds ratios for predictors of any wheezing (**A**) and recurrent wheezing (**B**) during the first year of life. Red markers indicate statistically significant predictors (95% CI excluding 1.0), and grey markers indicate non-significant predictors.

In the multivariable logistic regression model (Table 3; Figure 2A), NICU admission (aOR 2.65, 95% CI 1.49–4.73; *p* < 0.001) and parental allergic rhinitis (aOR 1.55, 95% CI 1.01–2.37; *p* = 0.043) remained independently associated with wheezing during the first year of life. Other variables were not independently associated with wheezing after adjustment. The model demonstrated good calibration (Hosmer–Lemeshow *p* = 0.753).

### 3.3. Predictors of Recurrent Wheezing During Infancy

Univariate associations with recurrent wheezing are shown in Table 2. NICU admission (OR 2.20, 95% CI 1.30–3.72; *p* = 0.003), parental eczema (OR 2.01, 95% CI 1.22–3.32; *p* = 0.006), parental allergic rhinitis (OR 1.68, 95% CI 1.06–2.67; *p* = 0.027), and child atopic dermatitis (OR 1.69, 95% CI 1.05–2.72; *p* = 0.032) were associated with increased odds of recurrent wheezing.

In the multivariable logistic regression model (Table 3; Figure 2B), NICU admission (aOR 2.34, 95% CI 1.21–4.56; *p* = 0.012), parental eczema (aOR 1.74, 95% CI 1.00–3.03; *p* = 0.049), and parental allergic rhinitis (aOR 1.67, 95% CI 1.00–2.78; *p* = 0.048) were independently associated with recurrent wheezing. The model demonstrated good calibration (Hosmer–Lemeshow *p* = 0.530).

## 4. Discussion

In this retrospective birth cohort of 594 infants followed through the first year of life in Riyadh, Saudi Arabia, 22.7% experienced at least one wheezing episode and 14.3% had recurrent wheezing, indicating that 63% of infants who wheezed experienced recurrent episodes. NICU admission was the strongest independent factor associated with both any wheezing (aOR 2.65, 95% CI 1.49–4.73) and recurrent wheezing (aOR 2.34, 95% CI 1.21–4.56). Parental allergic rhinitis was significantly associated with both outcomes, while parental eczema was specifically associated with recurrent wheezing. To our knowledge, this is the first birth cohort study from Saudi Arabia to examine predictors of infant wheezing during the first year of life.

NICU admission was the strongest factor associated with wheezing in our study, an association that remains underexplored because most birth cohorts have excluded NICU-admitted infants. Studies focused specifically on this group support our finding. In a Brazilian cohort of 277 preterm NICU graduates, Ramos et al. found that mechanical ventilation (OR 2.12, 95% CI 1.09–4.76) and prolonged oxygen therapy ≥ 15 days (OR 2.49, 95% CI 1.12–5.00) independently predicted recurrent wheezing, with a prevalence of 14.4%, similar in magnitude to our findings [17]. Hsu et al. reported that preterm infants requiring intensive respiratory support, a group overlapping with NICU graduates, had higher rates of preschool wheezing [18], and Sánchez García et al. linked viral infections acquired during NICU stay to recurrent wheeze in the first two years of life [19]. By including both preterm and term infants requiring NICU care, our study extends this literature to a broader spectrum of neonatal morbidity and to a Middle Eastern population. However, because NICU admission was analyzed as a single binary variable without information on its duration, indication, or the need for respiratory support, it is best interpreted as a marker of underlying neonatal morbidity rather than an independent causal factor, and residual confounding by neonatal respiratory morbidity cannot be excluded.

Parental allergic rhinitis was significantly associated with both wheezing outcomes in our cohort, consistent with international studies. Hallit et al. reported that maternal hay fever/eczema increased wheezing risk in French infants (aOR 1.13, 95% CI 1.02–1.24), while Fogaça et al. found a stronger association in Brazilian infants (OR 1.83, 95% CI 1.46–2.31) [9,11]. Our effect sizes fall within this range, supporting parental allergic rhinitis as a consistent predictor across diverse populations. Similarly, parental eczema was associated with recurrent wheezing in our study (aOR 1.74, 95% CI 1.00–3.03). Notably, parental asthma was not significant in our analysis despite being a strong predictor elsewhere, with maternal asthma reaching aOR 4.03 in Canadian infants [8]. This discrepancy may reflect differences in asthma prevalence or underreporting in our population.

Several factors examined in our study did not independently predict wheezing, despite associations reported elsewhere. Male sex was not a significant predictor, contrasting with consistent findings from Vanker et al. (IRR 1.41, 95% CI 1.16–1.72) and Fogaça et al. (OR 1.42, 95% CI 1.13–1.77). Gestational age, birth weight, child atopic dermatitis, and cesarean delivery also showed no significant associations. Environmental factors including household smoking, breastfeeding duration, and daycare attendance showed associations in univariate analysis but were attenuated in multivariable models. This is notable given strong evidence linking household smoking to infant wheezing in other cohorts, with Vanker et al. reporting IRR 1.55 for any household smoker and Gold et al. reporting RR 2.29 for maternal smoking [13,14]. The attenuation of these associations after adjustment may reflect confounding by NICU admission or socioeconomic factors. Alternatively, our modest sample size may have limited statistical power to detect these associations.

An additional consideration is that this cohort was born in 2020, during the COVID-19 pandemic. Public health measures, including reduced social mixing, daycare and school closures, and changes in healthcare-seeking behavior may have altered the circulation of common respiratory viruses, including respiratory syncytial virus and rhinovirus, and may therefore have influenced the incidence of wheezing during the first year of life [20,21]. This context should be considered when interpreting the observed prevalence and may limit comparability with cohorts recruited during non-pandemic periods.

## 5. Conclusions

This study has several strengths, including being among the first birth cohorts from Saudi Arabia to examine infant wheezing predictors and the inclusion of NICU-admitted infants often excluded elsewhere. We assessed a comprehensive range of risk factors using standardized outcome definitions. However, this study has several limitations. First, the retrospective design with parent-reported outcomes may have introduced recall bias and outcome misclassification, as infant wheeze can be difficult to distinguish from other forms of noisy breathing. Second, the 19.0% response rate raises the possibility of selection bias, as families with infants experiencing respiratory symptoms may have been more motivated to participate; because medical-record data were obtained only for respondents, a formal comparison of responders and non-responders was not possible, and the representativeness of the analytic sample could not be directly assessed. Third, the single-center setting may limit generalizability. Fourth, NICU admission was treated as a single binary variable, which does not capture the heterogeneity of neonatal morbidity. Finally, follow-up was limited to the first year of life, precluding inferences about wheezing persistence or asthma development.

Our findings may have clinical implications, but they should be interpreted cautiously. NICU admission should be viewed as a marker of underlying neonatal morbidity, particularly respiratory morbidity, rather than as an independent causal factor. Parental history of allergic rhinitis and eczema may also help identify infants who warrant further study. Future prospective multicenter studies with longer follow-up and more detailed characterization of NICU indications, respiratory support, and duration of admission are needed to confirm these associations and determine whether early predictors influence wheezing persistence and asthma development. In conclusion, NICU admission and parental atopy were associated with infant wheezing in this Saudi Arabian birth cohort. These findings are hypothesis-generating and warrant confirmation in prospective multicenter studies before informing changes in clinical practice.

## Figures and Tables

**Table 1:** Baseline characteristics of the cohort by wheezing status in the first year of life.

Characteristic	No Wheeze (n = 459)	Wheeze (n = 135)	*p*-Value
Male sex, n (%)	240 (52.3)	65 (48.1)	0.398
NICU admission, n (%)	65 (14.2)	41 (30.4)	<0.001
Cesarean delivery, n (%)	153 (33.3)	42 (31.1)	0.629
Child atopic dermatitis, n (%)	235 (51.2)	85 (63.0)	0.016
Parental asthma, n (%)	70 (15.3)	28 (20.7)	0.131
Parental allergic rhinitis, n (%)	180 (39.2)	69 (51.1)	0.014
Parental eczema, n (%)	90 (19.6)	38 (28.1)	0.034
Daycare attendance, n (%)	22 (4.8)	14 (10.4)	0.017
Household smokers, n (%)	108 (23.5)	34 (25.2)	0.692
Maternal employment, n (%)	138 (30.1)	44 (32.6)	0.576
Nearby pollution, n (%)	45 (9.8)	13 (9.6)	0.952
Reported home humidity, n (%)	9 (2.0)	7 (5.2)	0.063 *
Gestational age (weeks), Median (IQR)	39 (37–40)	39 (37–40)	0.846
Birth weight (grams), Median (IQR)	3100 (2700–3410)	3000 (2570–3500)	0.653
Breastfeeding duration (months), Median (IQR)	2 (1–3)	2 (1–3.5)	0.632

Note: Values are presented as n (%) for categorical variables and median (interquartile range, IQR) for continuous variables. *p*-values were calculated using the chi-square or Fisher’s exact test for categorical variables and the Mann–Whitney U test for continuous variables. The asterisk (*) indicates a *p*-value derived from Fisher’s exact test. NICU, neonatal intensive care unit.

**Table 2:** Univariable logistic regression of candidate predictors for any wheezing and recurrent wheezing during the first year of life.

	Any Wheezing (N = 594)			Recurrent Wheezing (N = 594)		
Predictor	Crude OR	95% CI	*p*-Value	Crude OR	95% CI	*p*-Value
Male sex	1.180	0.804–1.733	0.398	1.036	0.654–1.640	0.880
Gestational age (weeks)	0.942	0.883–1.005	0.069	0.955	0.884–1.031	0.234
Birth weight (per 500 g)	0.927	0.810–1.061	0.271	0.993	0.842–1.172	0.935
NICU admission	2.644	1.684–4.151	<0.001	2.202	1.304–3.716	0.003
Household smokers	1.094	0.701–1.706	0.692	0.834	0.477–1.458	0.524
Parental asthma	1.454	0.893–2.368	0.132	1.441	0.813–2.554	0.211
Parental allergic rhinitis	1.620	1.102–2.384	0.014	1.682	1.060–2.668	0.027
Parental eczema	1.606	1.034–2.494	0.035	2.009	1.216–3.320	0.006
Cesarean section	0.903	0.598–1.365	0.629	0.887	0.539–1.457	0.635
Daycare attendance	2.298	1.142–4.627	0.020	1.485	0.629–3.508	0.367
Home pets	0.942	0.343–2.587	0.908	0.895	0.260–3.078	0.860
Breastfeeding duration (months)	1.025	0.916–1.146	0.672	1.035	0.905–1.183	0.617
Child atopic dermatitis	1.620	1.092–2.404	0.016	1.688	1.047–2.722	0.032
Nearby pollution	0.980	0.512–1.877	0.952	1.112	0.525–2.356	0.782
Maternal employment	1.125	0.745–1.697	0.576	0.997	0.606–1.641	0.991

Note: Predictors meeting the screening threshold (*p* < 0.25) were carried forward to the multivariable model for the corresponding outcome. OR, odds ratio; CI, confidence interval; NICU, neonatal intensive care unit.

**Table 3:** Multivariable logistic regression of independent predictors for any wheezing and recurrent wheezing during the first year of life.

	Any Wheezing (n = 552)			Recurrent Wheezing (n = 552)		
Predictor	aOR	95% CI	*p*-Value	aOR	95% CI	*p*-Value
NICU admission	2.653	1.488–4.728	<0.001	2.344	1.205–4.560	0.012
Gestational age (weeks)	1.015	0.936–1.100	0.718	1.012	0.921–1.112	0.800
Parental asthma	1.410	0.834–2.383	0.200	1.259	0.673–2.354	0.471
Parental allergic rhinitis	1.551	1.014–2.371	0.043	1.671	1.004–2.782	0.048
Parental eczema	1.450	0.893–2.353	0.133	1.743	1.002–3.033	0.049
Child atopic dermatitis	1.312	0.855–2.014	0.214	1.343	0.801–2.254	0.263
Daycare attendance	1.774	0.801–3.929	0.158	—	—	—

Note: Variables with *p* < 0.25 in univariable analysis were entered into the multivariable model for each outcome. Daycare attendance met this screening threshold for any wheezing but not for recurrent wheezing and was therefore not included in the recurrent wheezing model. aOR, adjusted odds ratio; CI, confidence interval; NICU, neonatal intensive care unit.

## Data Availability

The data supporting the findings of this study are available from the corresponding author upon reasonable request. The data are not publicly available due to privacy and ethical restrictions.

## Supplementary Materials

The following supporting information can be downloaded at: https://www.mdpi.com/article/10.3390/healthcare14131996/s1.

## Abbreviations

The following abbreviations are used in this manuscript:

RSV	Respiratory Syncytial Virus
NICU	Neonatal Intensive Care Unit
KSUMC	King Saud University Medical City
EMR	Electronic Medical Record
OR	Odds Ratio
aOR	Adjusted Odds Ratio
CI	Confidence Interval
IQR	Interquartile Range
SPSS	Statistical Package for the Social Sciences
IBM	International Business Machines Corporation
RR	Relative Risk
IRR	Incidence Rate Ratio
aHR	Adjusted Hazard Ratio
Th2	T-helper Type 2
WHO	World Health Organization
IRB	Institutional Review Board
ORF	Ongoing Research Funding Program
EISL	International Study of Wheezing in Infants
