# Supplementary material for: Perinatal and Parental Predictors of Wheezing in the First Year of Life: A Saudi Arabian Birth Cohort Study
Source: Healthcare (Basel). 2026 Jul 5;14(13):1996. doi: 10.3390/healthcare14131996 (PMC13362337; doi:10.3390/healthcare14131996)
Supplement: Supplementary file 1 [file healthcare-14-01996-s001.zip › healthcare-4365622-supplementary.pdf]

Wheezing

مدى انتشار الأزيز التنفسي لدى الأطفال في أول سنة من العمر في منطقة الرياض

Prevalence of wheezing during the first year of life in Riyadh, Saudi Arabia

* 1. الموافقة على المشاركة في استبيان

عنوان البحث: مدى انتشار الأزيز التنفسي لدى الأطفال في أول سنة من العمر في منطقة الرياض

نحن مجموعة بحثية من كلية الطب (جامعة الملك سعود) بالرياض نعمل على دراسة بعنوان "قياس مدى انتشار الأزيز التنفسي لدى الأطفال في أول سنة من العمر في منطقة الرياض "

الغرض من هذه الدراسة هو تحديد مدى انتشار الأزيز التنفسي لدى الأطفال في أول سنة من العمر، وكذلك تحديد العوامل التي تساهم في زيادة نسبة الأزيز التنفسي. ستساعدنا البيانات في إكمال الدراسة مع العلم إن جميع المعلومات الشخصية سرية و لن يتم الإفصاح عنها بالدراسة.

يستغرق إكمال الاستبيان 3-5 دقائق فقط، ويمكنك الانسحاب من البحث في أي وقت.

أوافق على استخدام المعلومات في الدراسة البحثية ونشر النتائج

We are a research group from the College of Medicine at King Saud University in Riyadh, working on a study entitled “Prevalence of wheezing during the first year of life in Riyadh, Saudi Arabia”.

The purpose of the study is to determine the prevalence of wheezing in infants during the first year of their life in Riyadh in infants and investigate multiple factors that contribute to wheezing episodes in infants during the first year of life.

The data will help us to complete the study, knowing that all personal information is confidential and will not be disclosed in the study.

The survey only takes 3-5 minutes to complete, and you can opt-out of the research at any time.

I agree to participate in this survey, and the information to be utilized for scientific research purposes.

للتواصل (CONTACT)

د.ناصر الحربي (Dr.Nasser Alharbi)
0508804467
nalharbi@ksu.edu.sa

- ○ أوافق على المشاركة في الاستبيان
  Agree to participate in the survey
- ○ لا أوافق على المشاركة في الاستبيان
  Not agreeing to participate in the survey

Wheezing

* 2. الشخص الذي يقوم بتعبئة الاستبيان Person who will fill out data:

- ◯ Mother الأم
- ◯ Father الأب
- ◯ شخص آخر (يرجى التحديد)
  Other (please specify)

[ ]

* 3. اسم الطفل
Child's first name

[ ]

* 4. عمر الطفل: .... شهر
Age (in months)

[ ]

* 5. جنس الطفل
Sex

- ◯ Femal أنثى
- ◯ Male ذكر

* 6. ماهي جنسية الطفل؟
Child nationality?

- ◯ Saudi سعودي
- ◯ جنسية اخرى (يرجى التحديد)
  Other nationality (please specify)

[ ]

* 7. رقم التواصل (جوال)
Your Mobile number

[ ]

8. وزن الطفل عند الولادة اذا كنت تعرفه:
كيلو و .... غرام (مثال ٣ كيلو و ١٠٠ غرام = 3100 ....)
Birth weight in kilograms and grams, if you know
(example 3 kilograms 100 grams = 3100)

[ ]

9. غرام ……………… كيلو و …………… وزن الطفل حاليًا إذا كان معلوم:
(مثلا 5 كيلو و 200 غرام = 5200)
current weight (if you know) Kilograms and Grams (Example 5 Kg and 200 grams = 5200)

10. سم ……………… طول الطفل عند الولادة إذا كان معلوم:
Birth height if known: Cm

11. سم ………………… طول الطفل حاليًا إذا كان معلوم:
Current height if known: Cm

* 12. هل سبق لطفلك دخول العناية المركزة بعد الولادة؟
Did your child' admit to NICU

- Yes نعم
- No لا

* 13. مكان اقامة الطفل (المدينة): ………………………..
City

- Riyadh الرياض
- مدينة أخرى (يرجى تحديدها)
  Other city (please specify)

* 14. هل كان لدى طفلك أزيز (صفير) في الصدر أو التهاب القصبات خلال السنة الأولى من العمر؟
Has your baby had wheezing in the chest or bronchitis or whistling during his/her first 12 months of life?

- Yes نعم
- No لا

Wheezing

* 15. كم كان عدد حالات الأزيز في أول 12 شهر ؟
How many episodes of wheezing in the chest (bronchitis or whistling) did your baby have during the first year?

- 0
- أقل من3
  Less than 3 episodes
- من 3 إلى 6
  3 to 6 episodes
- أكثر من 6
  More than 6 episodes

* 16. كم كان عمر الطفل (بالأشهر) عند حدوث أول نوبة أزيز (صفير) في الصدر؟ .... شهر
At what age did your baby first have an episode of wheezing in the chest (bronchitis or whistling)? At ……………….. months.

* 17. هل تم علاج طفلك بعلاجات لتوسيع القصبات الهوائية من خلال بخاخ أو رذاذ (مثل الفنتولين)؟
Has your baby been treated with inhaled medications to relieve chest wheezing (bronchodilators) via nebulizers or inhalers (sprays)? For example salbutamol, aerolin, berotec, bricanyl.

- Yes نعم
- No لا
- I do not know لا أدري

* 18. هل تم علاج طفلك ببخاخات الكورتيزون ؟
Has your baby been treated with inhaled corticosteroids (cortisones in spray form)? For example symbicort, flixotide, seretide, clenil, beclosol, budesonide, busonid, pulmicort, beclometasone, fluticasone.

- Yes نعم
- No لا
- I do not know لا أدري

* 19. هل تم علاج طفلك بعلاجات مضاد الليوكوتراين (السنقيولير) ؟
Has your baby been treated with antileukotrienes? Singulair.

- Yes نعم
- No لا
- I do not know لا أدري

* 20. خلال السنة الأولى من عمر الطفل كم عدد المرات التي استيقظت فيها من النوم بسبب كحة الطفل أو الأزيزالصدري؟
During the last 12 months, how many times have you woken up during the night because your baby was coughing or had a wheezing chest?

- Never ولا مرة
- Rarely (less than once a month) مرة في الشهر تقريبا
- Sometimes (some weeks of some months) (بعض الأوقات) اكثر من مرة في الشهر
- Frequently (2 or more per week, almost every month) (كثيرا) مرتين في اسبوعيا بشكل مستمر

* 21. خلال ال12 شهر الأخيرة هل كان هنالك أي حالة شديدة من الأزيز الصدري جعلتك تذهب بالطفل للطوارئ او المستشفى؟
During the last 12 months, has the wheezing (whistling) in your baby's chest been so strong that you have had to seek emergency services (hospital, clinic, or health center)?

- No لا
- نعم (كم مرة)؟
  Yes (please specify how many times)?

* 22. خلال السنة الأولى هل تعرض طفلك لحالة شديدة من الأزيز أو الصفير أدى لصعوبة في التنفس؟
During the last 12 months, has the wheezing (whistling) in your baby's chest been so intense that you have felt it caused great difficulty in breathing (shortness of breath)?

- Yes نعم
- No لا

* 23. هل سبق إدخال طفلك للمستشفى بسبب التهاب القصبات الهوائية (أو التهاب بالشعب الهوائية) ؟
Has your baby been admitted to the hospital for bronchitis?

- Yes نعم
- No لا

* 24. هل سبق تشخيص طفلك بالربو ؟
Has a doctor ever told you your baby has asthma?

- Yes نعم
- No لا

Wheezing

* 25. هل سبق تشخيص طفلك بالالتهاب الرئوي؟
Has your baby ever had pneumonia?

- ◯ Yes نعم
- ◯ No لا

* 26. هل سبق إدخال طفلك للمستشفى بسبب إلتهاب رئوي؟
Has your baby ever been admitted to the hospital for pneumonia?

- ◯ Yes نعم
- ◯ No لا

* 27. هل يوجد في المنزل أي مدخن ؟
Does anyone smoke inside your house (father, mother, grandparents, uncles)?

- ☐ Father الاب
- ☐ Mother الأم
- ☐ None لا أحد
- ☐ آخرون (يرجى التحديد)
  Other (please specify)

____

* 28. هل سبق لأم الطفل التدخين أثناء الحمل؟
Did the mother of your baby smoke during pregnancy?

- ◯ Yes نعم
- ◯ No لا

* 29. هل يوجد في العائلة أشخاص مصابون بالربو؟
(اختر كل ما ينطبق)
Does your baby have any family members with asthma?
(Choose all that apply)

- ☐ Mother الأم
- ☐ Father الأب
- ☐ Brother or sister الاخوة والاخوات
- ☐ Grandparents الجد أو الجدة
- ☐ No one لا يوجد أحد

* 30. هل يوجد في العائلة أشخاص مصابون بحساسية الأنف؟
Does your baby have any family members with hay fever or allergic rhinitis?

- [ ] Mother الأم
- [ ] Grandparents الجد أو الجدة
- [ ] Father الأب
- [ ] No one لا يوجد أحد
- [ ] Brother or sister الاخوة والاخوات

* 31. هل يوجد في العائلة أشخاص مصابون بحساسية الجلد (الاكزيما)؟
Does your baby have any family members with skin allergies (allergic dermatitis)?

- [ ] Mother الأم
- [ ] Grandparents الجد أو الجدة
- [ ] Father الأب
- [ ] No one لا يوجد
- [ ] Brother or sister الاخوة والاخوات

* 32. هل كانت الولادة بعملية قيصرية؟
Was your baby delivered by cesarean section?

- ( ) No لا
- ( ) Yes نعم

* 33. هل يذهب الطفل الى الحضانة ؟
Has your baby attended daycare this year?

- ( ) No لا
- ( ) Yes نعم

* 34. كم مرة يأكل الطفل من خارج المنزل؟
How often do you feed your baby any of the following products (not home-made): yogurt, pudding, chips, chocolate, soda, fruit juice in bottle or box, artificial jam...

- ( ) Never أبدا
- ( ) Every day كل يوم
- ( ) Once a week مرة بالأسبوع
- ( ) Once a month مرة بالشهر

* 35. هل تعيش حيوانات في المنزل؟
Do you currently keep pets at home? (dog, cat, bird, rabbit)

- [ ] Cat نعم, قطط
- [ ] Dog نعم, كلاب
- [ ] Bird نعم, طيور
- [ ] None لاتوجد حيوانات منزلية
- [ ] نعم, غيرها
  Other (please specify)

* 36. أعلى مستوى تعليمي للآم ؟

Mother's level of education

- Illitrate غير متعلم
- Elementary school ابتدائي
- Intermediate school متوسط
- High school ثانوي
- University جامعي
- Master دراسات عليا

* 37. أعلى مستوى تعليمي للأب ؟

Father's level of education

- Illitrate غير متعلم
- Elementary school ابتدائي
- Intermediate school متوسط
- High school ثانوي
- University جامعي
- Master دراسات عليا

* 38. كم عدد أشهر الرضاعة الطبيعية فقط؟ (بدون حليب صناعي أو أغذية أخرى يشمل العصير)؟

For how many months did you only breastfeed your baby (without giving juices, baby food, or any other kind of milk)? ………. Months

- Never نهائيا
- Less than one monthأقل من شهر
- Less than 3 months أقل من ثلاثة أشهر
- months 3-6 أشهر
- months 6-9 أشهر
- months 12 شهرا

* 39. كم مرة اصيب الطفل بالزكام أو الانفلونزا خلال السنةالأولى من العمر؟

How many colds (episodes of sneezing, coughing and nasal discharge with or without fever) did your baby have during his first year of life? (Write the number in space. ……… episodes.

* 40. كم عمر الطفل حينما تعرض للزكام الأول بالأشهر؟

How old was your baby when he/she got his/her first cold? (Write age in months in the space. ………… months

* 41. هل عانى الطفل من أي حساسة في الجلد في السنة الأولى؟

Did your baby have or does he/she still have any skin allergies during his/her first year of life (itchy red spots on the skin, allergy to diaper, allergy to mosquito bites, to food, metals, etc..)?

- No لا
- Yes نعم

* 42. هل هناك تلوث في البيئة المحيطة (مصانع أو عوادم سيارات أو غيره)

Do you think the place where you live has atmospheric pollution (factory smoke, road traffic, etc)

- A great deal كثير جدا
- A lot كثير
- A moderate amount متوسط
- A little قليل
- None at all لا يوجد

* 43. هل توجد رائحة رطوبة عالية في المنزل؟
Are there mold (mildew) or humidity stains in your house?

- No لا
- Yes نعم

* 44. كم عدد الأشخاص (بالغين و أطفال) الذين يسكنون في نفس المنزل مع الطفل؟
How many people (adults and children) currently live at your home?

* 45. كم عدد اخوة و اخوات الطفل في المنزل؟
How many siblings does your baby have?

* 46. هل الأم تعمل؟
Do you (the mother) currently have paid work?

- Yes نعم
- No لا

* 47. هل سبق أن أخذ الطفل كورتيزون عن طريق الفم أو الوريد؟
Has your baby been treated with oral corticosteroids (predsim, prelone, decadron)?

- Yes نعم
- No لا
- I do not know لا أدري

* 48. هل اكمل الطفل جميع التطعيمات المطلوبة في عمره الحالي؟
Is your baby’s immunization up to date (corresponding to the first year)?

- Yes نعم
- No لا
